# Therapeutic effect of nerve growth factor on canine cerebral infarction evaluated by MRI

**DOI:** 10.18632/oncotarget.23345

**Published:** 2017-12-16

**Authors:** Yong Wang, Hui Zhang, Zhe Wang, Huaijun Liu, Xin Tian, Jian Yu, Chaoxu Chen

**Affiliations:** ^1^ Department of Radiology, The Second Hospital of Hebei Medical University, Shijiazhuang, Hebei Province 050000, China; ^2^ Department of Radiology, Hebei General Hospital, Shijiazhuang, Hebei Province 050051, China; ^3^ Department of Computer Science, Clinical College of Hebei Medical University, Shijiazhuang, Hebei Province 050031, China

**Keywords:** nerve growth factor, canine middle cerebral artery occlusion, magnetic resonance imaging, diffusion-weighted imaging, perfusion-weighted imaging

## Abstract

To explore therapeutic effect of nerve growth factor (NGF) on cerebral infarction by establishing canine middle cerebral artery occlusion (MCAO) infarct model. The magnetic resonance imaging (MRI) technology was used to study effects of NGF on cerebral infarction, and the results of MRI indexes (such as diffusion-weighted imaging (DWI) and perfusion-weighted imaging (PWI)) were compared with the results of pathology, cell biology and molecular biology. The clinical manifestations of the canine infarction model treated by NGF were significantly improved within 7 days compared with control group. The therapeutic evaluation of NGF effect could be determine by canine cerebral infarction treated by NGF within 6 hours according to DWI and PWI. From 6 hours to 7 days, therapeutic evaluation of NGF could be determine by T1WI, T2WI and FLAIR. DWI and PWI could find the change of cerebral ischemia at the early stage, provide advantages for qualitative diagnosis of early-stage cerebral infarction and observation of efficacy in early treatment, initially showing that their great potential for NGF role on cerebral ischemia and mechanism.

## INTRODUCTION

Cerebral infarction is a common type of cerebrovascular disease, and is resulted from hypoxia, cerebral blood circulation disorder, and ischemia, which is a likely cause of age-related cognitive decline and dementia because of thrombosis [1, 2]. As the ranked first disabling disease and second fatal disease in China, cerebral infarction has seriously effect on patient’s quality of life [3]. The treatment methods for cerebral infarction have been developed for many years, but recovery of nerve tissue rehabilitation is difficult and delayed because a lot of neurons die during cerebral infarction.

Nerve growth factor (NGF) is a protein primarily responsible for the differentiation and survival of neurons in the peripheral nervous system [4], so NGF has been one of neuroprotective agents for treating cerebral infarction [5–7] and ischemic brain [8, 9]. The situation of focus recovery was observed and evaluated after cerebral infarction treated by NGF and other neuroprotective agents, which needed more reliable objective indicators. Using imaging methods, especially magnetic resonance imaging (MRI) technology can directly observe changes of scope and extent in focus under the role of NGF [10]. In addition to routine T1WI, T2WI and FLAIR scans, MRI includes diffusion-weighted imaging (DWI) and perfusion-weighted imaging (PWI) [11]. DWI is sensitive to Brownian motion of water molecules and can detect the apparent diffusion coefficient (ADC) of living tissue water. While PWI can be used to analyze ischemic blood flow changes, and can provide brain tissue blood supply information by semi-quantitative way, which indirectly explain the survival of neurons and glial cells. In the hyperacute stage of cerebral infarction, DWI and PWI showed varying degrees of ischemic focus [12], which created a change for us to evaluate image performance of ischemic focus at different development stages by using DWI and PWI.

Selection of model animals included canines, pigs and monkeys, whose developmental system and dietary structure were similar to those of humans and can be used in the production of ischemic models [13, 14]. But canines were more available and cheap than two others, the canine model of acute cerebral infarction caused by middle cerebral artery occlusion was established in this study. After NGF was injected into cerebral surrounding focus, the MRI indexes were compared with the results of pathology, cell biology and molecular biology between the treatment and control groups at different time periods.

## RESULTS

### Results of MRI

#### Regular scanning

**Figure 1:**
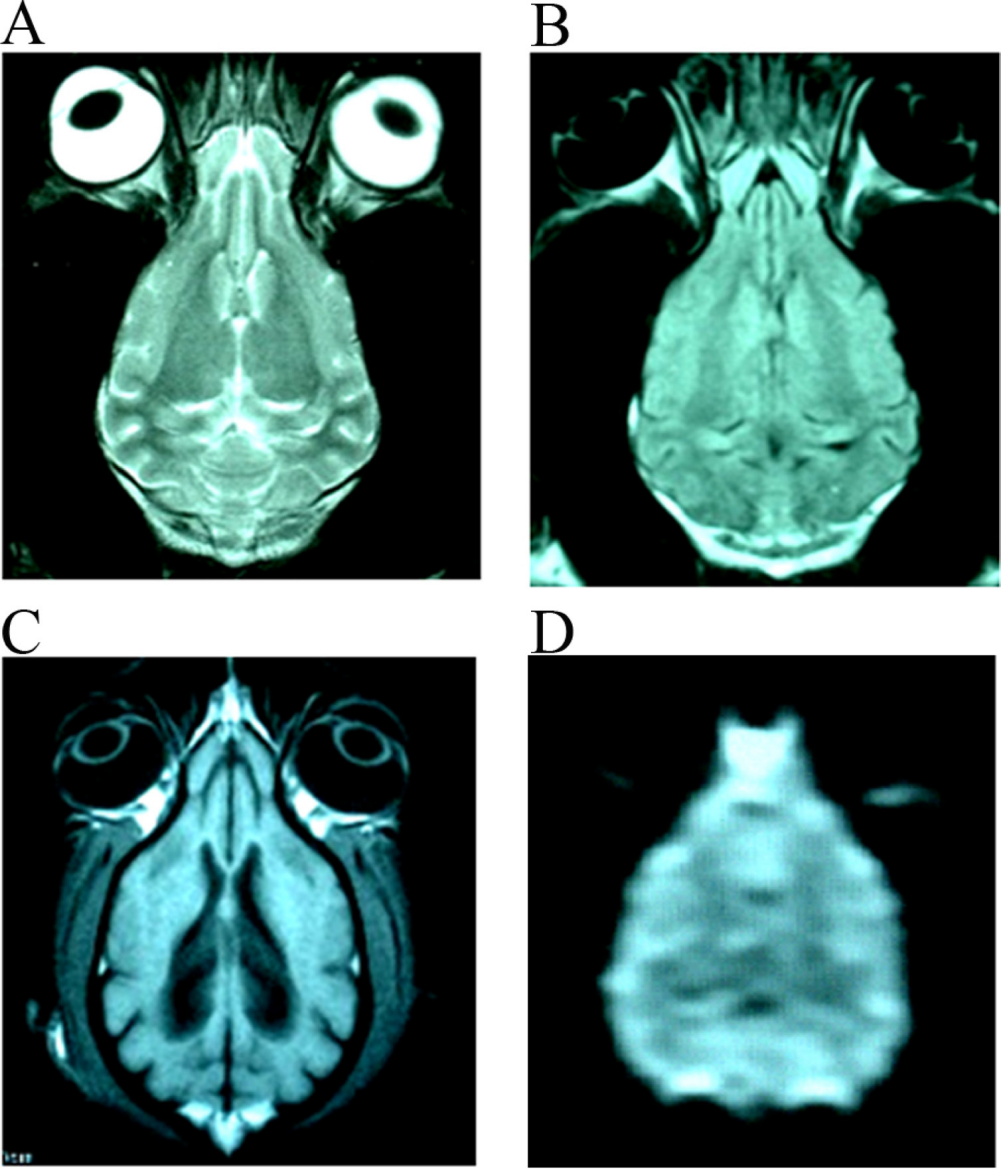
The normal images of T2WI (**A**), FLAIR (**B**), T1WI (**C**) and DWI (**D**) from the same slice of canines’ brain.

After stroke, the signal intensity of lesion in DWI images were increased gradually in those without treating canines at 0.5 h, 6 h and 24 h. In those treated canines, the signal intensity of DWI images at different time points also increased, but were not so evident like those untreated canines (Supplementary Figure 1). The images (DWI, T1WI, T2WI and FLAIR sequences) of infarction focus in group E at each time point were more serious than that of groups A-D at the range and signal intensity of the focus. There was no abnormal MRI scan in the F group at each time point (Figure 1).

#### Changes of ADCR

By comparing each other, it was found that the ADC values obtained form each time point after surgery were lower than those of the opposite side normal tissue (ADCR < 1) and approached to the opposite side after 3 months. In this process, ADC value changes of different treatment groups and different scan time were not the same. Table 1 summarized the variance analysis results for ADCR in different treatment groups.

**Table 1:** Analysis of variance analysis results of ADCR between groups at different ischemic time

Ischemic time	NGF treatment	NaCl control	*F* value	*P* value
3 hours	0.4554 ± 0.0770	0.4771 ± 0.0567	0.269	>0.05
6 hours	0.6046 ± 0.0586	0.5576 ± 0.0588	1.591	>0.05
24 hours	0.8253 ± 0.0332	0.9071 ± 0.0410	12.244	<0.01
7 days	0.7955 ± 0.0564	0.8820 ± 0.0538	9.692	<0.01
3 months	0.8140 ± 0.0463	0.8691 ± 0.0482	10.836	<0.01

#### Changes in HLA%

The diffusion abnormal area rapidly expanded after 0.5 to 6 hours of MCAO, the main part was in right middle cerebral artery. The increased average rate of HLA% for diffusion abnormal area was 6.22%/h. At 6–12 hours, increased average rate of HLA% for diffusion abnormal area was 0.52 %/h. At 12–24 hours, relatively rapid period of HLA% was found with increased average rate of 3.66 %/h. Also, the increased abnormal range was mainly located in the cortical region of the middle cerebral artery. The levels of HLA of the NGF groups (groups A-D) in the DWI were lower than those in group E at the same time point from 6 hours to 3 months after surgery. HLV% of group D at 3 months was compared with group E, as shown in Supplementary Figure 2.

#### Perfusion imaging results

As show in Supplementary Figure 3, perfusion curve of infarct focus was higher than contralateral normal tissue in group E, while in group B, perfusion curve of infarct focus was similar with contralateral normal tissue. The rCBV in successful experimental animal models (such as group E) within 6 hours was decreased, but did not reach at 0, and generally decreased to the corresponding opposite side area of 14.22%; change of rCBV was slowly fluctuated at 1 week, and was only 17.72% of the corresponding opposite side area. During 2 weeks to 3 months, the change was enhanced and increased significantly. During this time period, the highest recovery was 72.46 % of the corresponding opposite side area. When the rCBV was increased, the MTT time was also slowly reduced accordingly. The rCBV in group D was decreased within 6 hours, and was 12.85 % of the corresponding opposite side area, but there was no significant difference compared with control group (*P* > 0.05). From 6 hours to 7 days, group D showed more obvious recovery, the highest could be 30.46% of the corresponding opposite side area, which showed significantly different compared with group E (*P* < 0.01). From 2 weeks to 3 months, the rCBV in the focus region was increased to 86.32% of the corresponding opposite side area, and there was significant difference between two groups (*P* < 0.05).

#### Clinical manifestations

The neurological function impairment of the animals in the NGF treatment group (groups A-D) was mild and the recovery time was shorter, while neurological function impairment of group E was more serious, and recovery time was longer. The neurologic impairment scores were performed at 24 hours after surgery with an average score of 9.22 ± 1.25, indicating that clinical symptoms of all the surviving animals had been significantly recovered. Most of the animals had recovered in apparent normal state at 7 days after surgery with an average score of 3.67 ± 0.53. At 3 months after surgery, in addition to symptoms caused by a slight cerebral infarction, animals had been basically normal with an average score of 2.13 ± 0.26.

### Morphological observation

#### General observation

**Figure 2:**
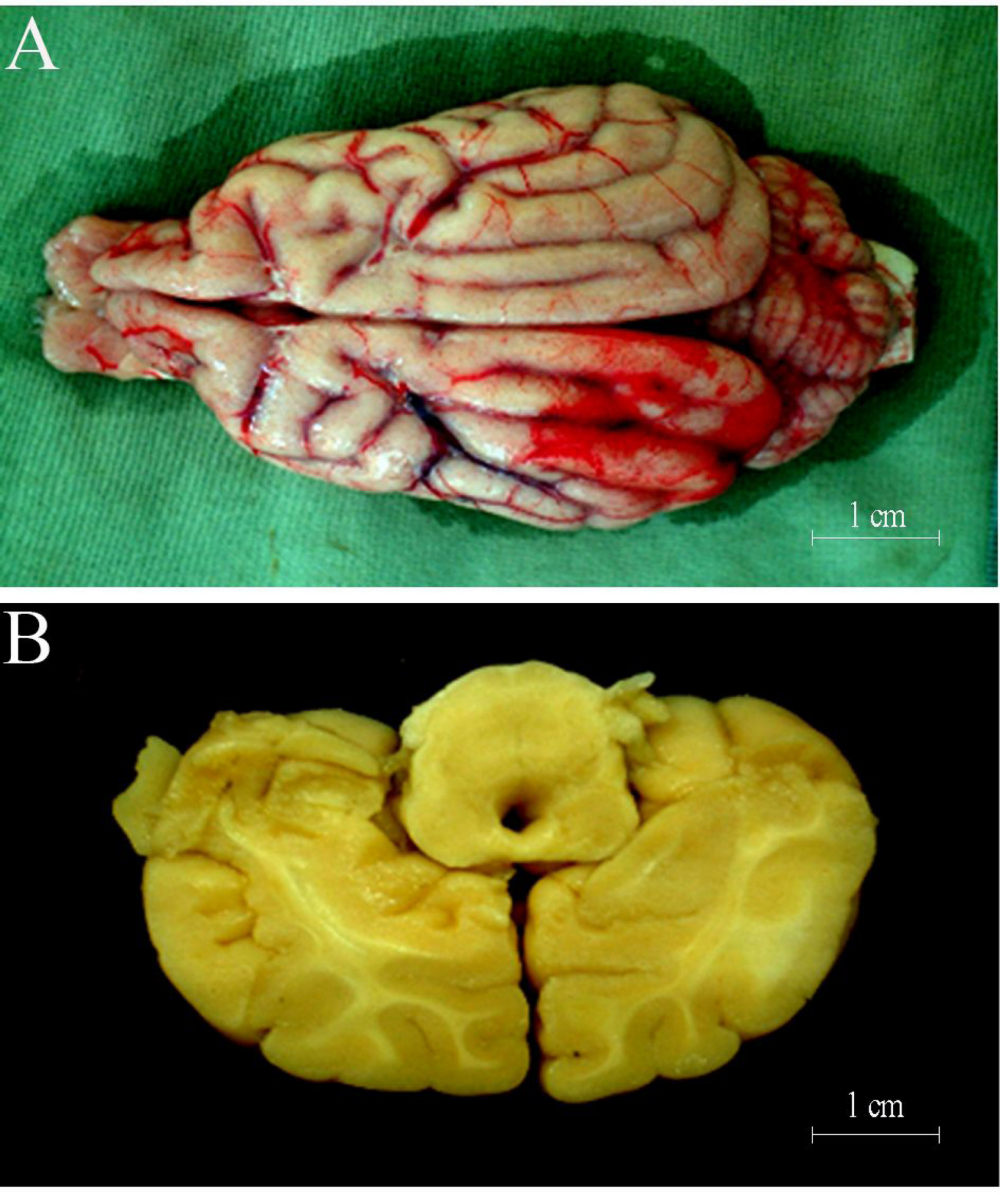
(**A**) 24 hours after cerebral ischemia, some microressels could be discerned on surface of cortex at the site of lesion; (**B**) 24 hours after cerebral ischemia, some lesion could be discerned on cortex.

Brain tissue swelling of group E could be found infarct side at 6 hours after MCAO surgery, and the midline was displaced to the opposite side at varying degrees. The peripheral vascular was changed to be thicker in the infarcted lesions at 6 hours after surgery (Figure 2A), and junction of cortex and medulla of the infarcted area was blurred at cross section (Figure 2B). The symptom of animals in groups A-D were milder than those in group E at different time points (Supplementary Table 2). The degree of brain tissue swelling and displacement degree in group A were milder than group E, and the midline was shifted to 0.16 cm. The size of softening focus in the brain tissue of group D was 3.52 cm × 1.88 cm × 1.32 cm, and softening focus in the E group was 2.64cm × 1.15 cm × 0.69 cm at 3 months.

### Light microscopy (HE staining)

**Figure 3:**
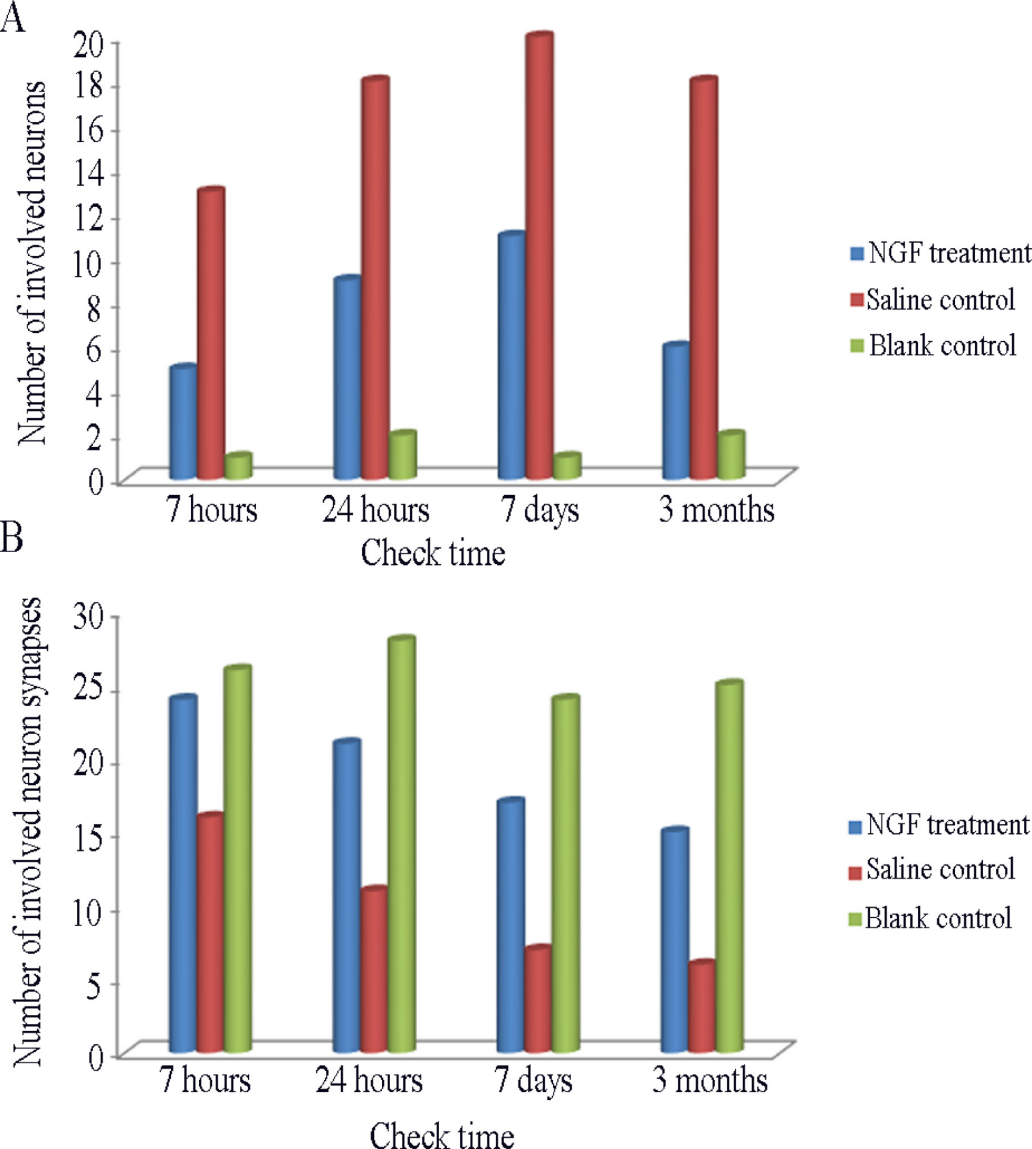
(**A**) Comparison of the number of involved neurons in per view (HE staining × 400) among different group. (**B**) Comparison of the number of involved neuron synapses in per view (Electron Microscope × 4000) among different group.

The results of HE staining in the MCAO animals treated with NGF were improved in different degree, and the neurons in the ischemic area of group A treated with NGF for 6 hours were moderate shrinkage, and the degree of karyopyknosis, gap of nerve fiber, and width of glial cells and microvascular gap were significantly narrower than infarction focus without NGF treatment (Supplementary Figure 4). As shown in Figure 3A, number of involved neurons in NGF treatment group were lower than group E, but higher than group F (*P* < 0.05). The acidophilic neurons in group B and C were increased by about 14% and 19% compared with the normal control group. Ghost cells and other abnormal cells were relatively small number in group B (0) and C group (5)

### Electron microscopic observation and synaptic counting

Due to without treatment, electron microscopy of E group showed only the natural changes in infarcts. At 6 hours after surgery, neurons was slightly swollen, intracellular mitochondrial swelling was obvious, and the internal chamber was expanded. At 24 hours after surgery, neuron was significantly swollen, the number of organelles were decreased, and mitochondrial swelling was more obvious (Supplementary Figure 5). At 7 days after surgery, neuron swelling at edge of infarction focus was alleviated, and the morphological changes of mitochondria, Golgi complex and rough endoplasmic reticulum were improved. At 3 months after surgery, a large number of oligodendrocytes were observed and the residual neurons was less, and the number of synapses were decreased significantly.

**Figure 4:**
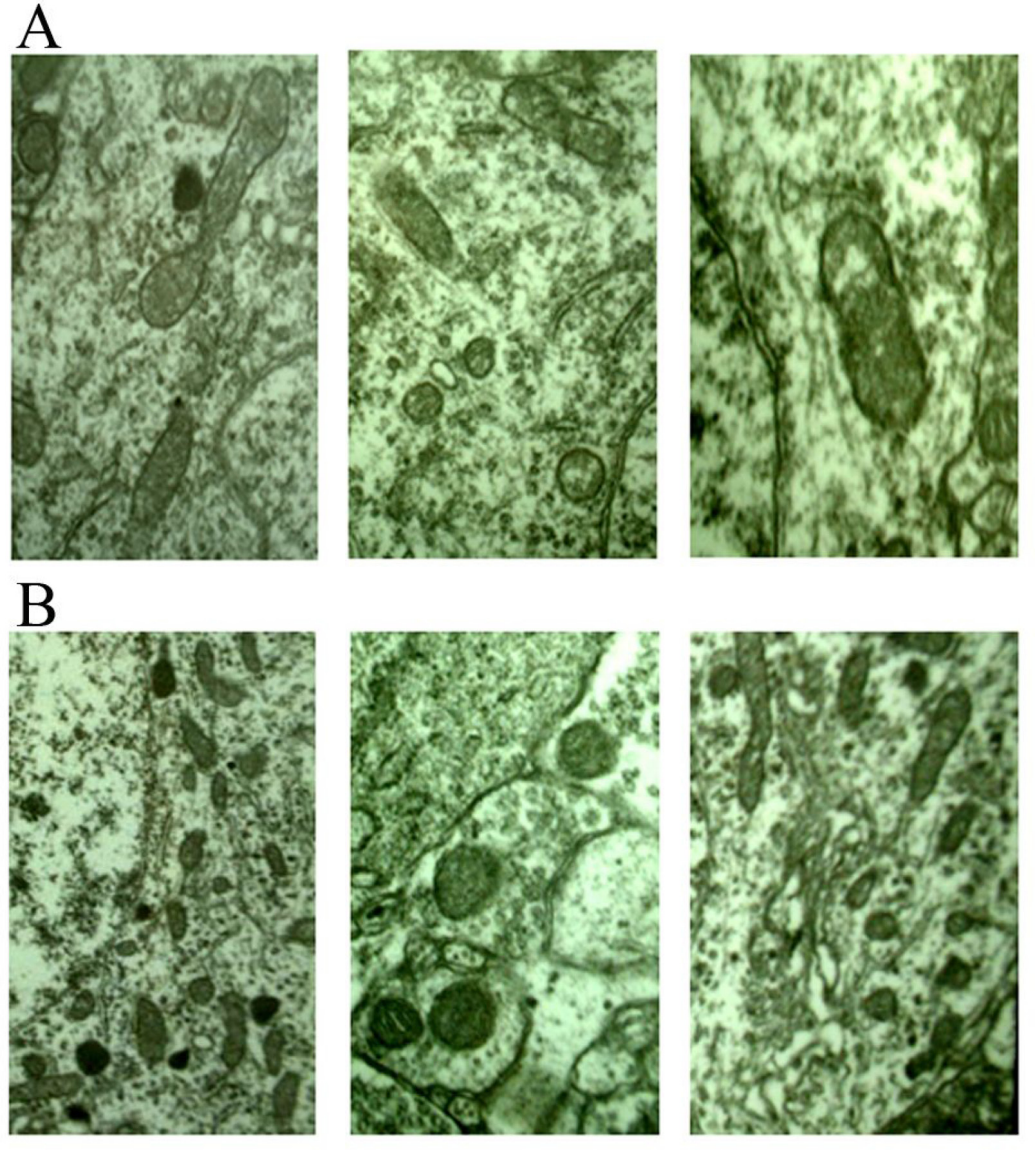
(**A**) 6 hours after stroke, mitochondrion edema was seen (×20000) from different views; (**B**) 24 hours after stroke, mitochondrion edema was severe, the number of lipofascin paoticles were decreased (×20000) from different views.

The number of involved neuron synapses in in NGF treatment group were lower than group E, but higher than group F (Figure 3B). The manifestation of neurons in groups A-D after NGF treatment was significant higher than that in group E, and the number of viable neurons in group D were significantly increased in the same magnification field, and the axons and synapses of neurons were also significantly increased (Figure 4). Synaptic count of NGF treatment groups (groups B-D) showed significant difference compared with group E (*P* < 0.01) (Table 2). There was also a significant difference between NGF treatment groups and group F (*P* < 0.01), while NGF treatment groups showed no significant difference (*P* > 0.05).

**Table 2:** Neural synapse of different group at different ischemic time*

Ischemic time	NGF treatment	NaCl control	*F* value	*P* value
6 hours	12.25 ± 1.80	12.36 ± 1.75	2.173	>0.05
24 hours	12.19 ± 2.16	11.28 ± 1.66	11.528	<0.01
7 days	13.08 ± 2.03	12.36 ± 2.31	8.511	<0.01
3 months	13.31 ± 2.35	12.20 ± 2.03	10.661	<0.01

^*^Counts/×8000.

### Immunopositive cell count of immunohistochemical staining

**Figure 5:**
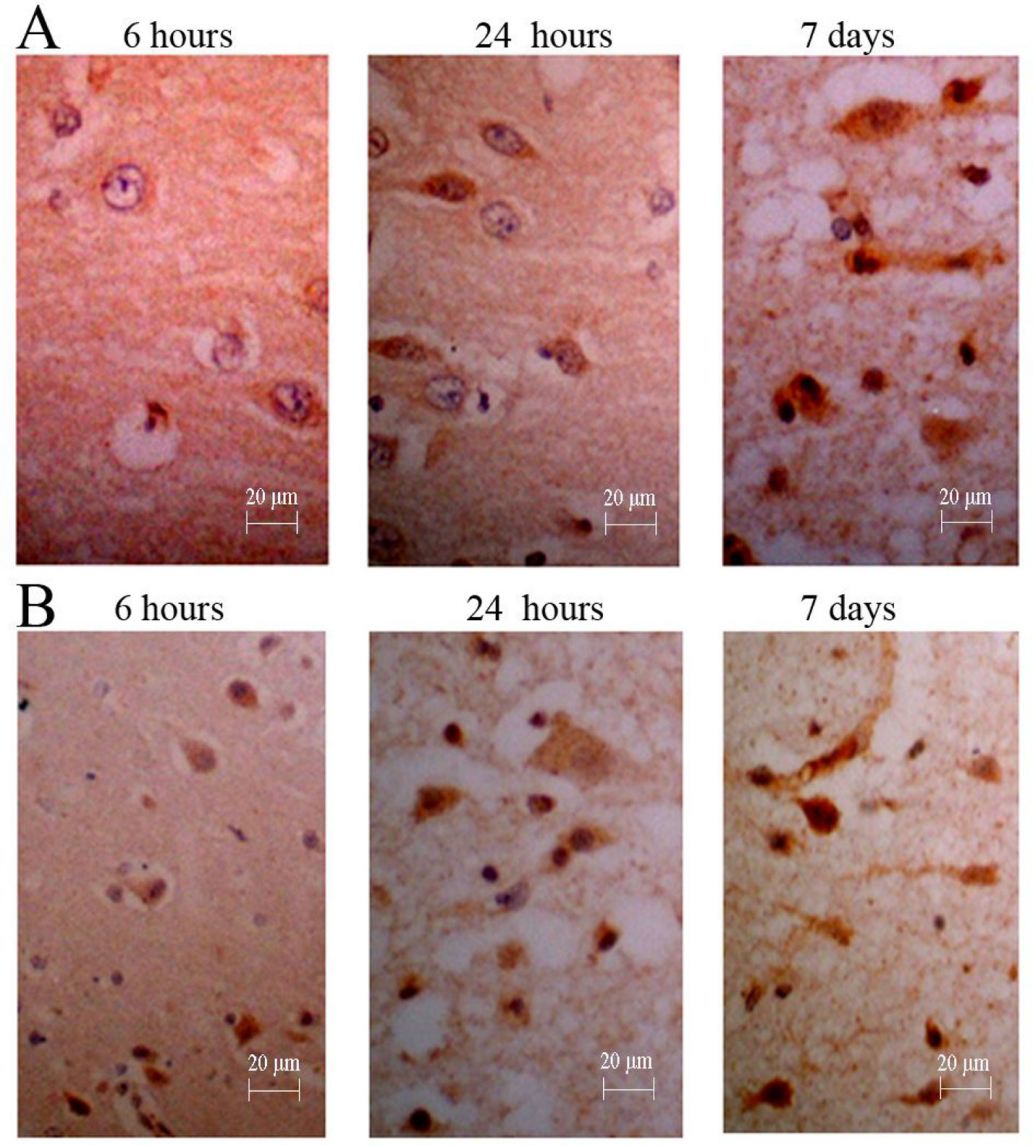
Immunohistochemistry for NGF of canines’ brain (**A**) The number of positive cells was increased gradually, and the degree of straining also was increased gradually; (**B**) The counts and degree of straining were more in those treated canine from group D (NGF treatment for 3 months).

The immunopositive cells in each group treated with NGF were basically consistent with those of group E at expression time-histories. The mild increase of immunopositive cells (3–17 per view) was observed in group A at 6 hours after surgery, and the immunopositive particles were stained lightly. At this time, significant increase in immune-positive cells (34–78 per view) could be found in group B, immunopositive particles were stained deeply (Figure 5). In group C, the number of immunopositive cells (28–56 per view) was slightly less than that of group B, and the staining of immunopositive particle was slightly lighter. The number of immunoreactive cells (2–12 per view) and degree of staining of immunopositive particle in group D were similar to those of group F. In the visual field, a small amount of immune-positive cells and stained immunopositive particles could be seen in group F (0–6 per view).

After results of immunopositive cell counts were analyzed by ANOVA, groups B and C showed significant differences compared with group E (*P* < 0.01) (Table 3). The groups B and C showed significant differences compared with group F (*P* < 0.01). There was no significant difference among group A, D and group E (*P* > 0.05).

**Table 3:** NGF immunostained cells of different group at different ischemic time*

Ischemic time	NGF treatment	NaCl control	*F* value	*P* value
6 hours	30.20 ± 3.08	30.40 ± 2.70	0.048	>0.05
24 hours	40.79 ± 3.03	33.10 ± 3.67	13.401	<0.01
7 days	51.68 ± 3.47	42.25 ± 3.08	11.673	<0.01
3 months	58.61 ± 4.34	55.20 ± 4.09	1.628	>0.05

^*^Counts/×400.

## DISCUSSION

NGF is one of the most important biological activators of the nervous system, which can affect the survival and differentiation of certain neurons in the central nervous system [15]. In the natural state, ischemic brain damage can lead to an increase in endogenous NGF expression [16], and this can also be validated in this study. After cerebral ischemia, expression of endogenous NGF and its receptor were increased [17, 18]. Hsu *et al.* [19] made the right middle cerebral artery occlusion for 30 minutes and then open, the expression of cortical NGF was increased at 4 hours after ischemia, expression was inhibited at 1 day to 3 days, but second peak was found at 1 week to 4 weeks. Thus it could be seen. Whether it was short-term cerebral ischemia or prolonged ischemia, expression of NGF could be increased. However, the amount of endogenous NGF production was very small, which was far from meeting the need for healing of damaged neurons. Therefore, it was necessary to give exogenous NGF to reduce the infarct size caused by ischemia and promote neuronal recovery and regeneration [20]. NGF promoted the growth and regeneration of nerve fibers and the formation of synapses, and the synaptic counts were significantly increased under electron microscope. These results suggested that NGF could effectively protect damaged neurons and promote their recovery and regeneration, which were consistent with the results of this study.

The comparison of MRI, cell biology and molecular biology of the ischemic cerebral infarction in canis familiaris at different time points showed that the neuronal protection and repair role of NGF were mainly found within 24 hours after MCAO. During this time period, the DWI on the early treatment group showed that the abnormal signal range was relatively narrowed at each time point compared with the control group, while the descend range of ADC values was reduced; PWI showed that descend range of CBV and increase range of MTT in the treatment groups were smaller than the saline group and control group. These results indicated that DWI, PWI and T2WI had the protective and repair effects of NGF on penumbra damaged neurons in canine with cerebral infarction at different times. The HE staining and immunohistochemical staining results and electron microscopy ultrastructural observation had confirmed these results.

It had been shown that DWI was significantly earlier than conventional T2WI in acute cerebral infarction, and the ADC value of ischemic site was significantly lower than that of normal brain tissue, which was helpful for the early diagnosis of ischemic cerebral infarction, estimated size of infarct range, efficacy evaluation of treatment and prognosis assessment of the disease [21]. Because DWI was sensitive to brain tissue ischemia, and made it possible to become early imaging evaluation means for NGF and other neuroprotective agents in the treatment of cerebral infarction. In this study, early imaging evaluation means DWI and the ADC value measured by DWI could clearly determine the range and extent of ischemic cerebral infarction in canine, compare the difference in the image caused by treatment, and thus indirectly determine the repair degree of NGF on individual cerebral infarction and the efficacy of NGF.

The increase of DWI image signal and the decrease of ADC value in the hyperacute stage of cerebral infarction reflected the disorder of energy metabolism in the period of “cytotoxic edema”. Cytotoxic edema and vasogenic edema belonged to two stages of ischemic brain edema, but there was no uniform standard in the distinction between two stages. When at 0.5 hour after MCAO, DWI had shown the high signal. At 3 hours after MCAO, electron microscopy showed only mitochondrial swelling and degranulation of the rough endoplasmic reticulum. At 6 hours of ischemia, mitochondria and rough endoplasmic reticulum swelling were significantly increased. Close connection between the endothelium had been destroyed, at this time, vasogenic edema had been replaced by cytotoxic edema, and edema signal was spread more widely. At this stage, the signal of focus by DWI was quickly increased, while the ADC value and ADCR were gradually decreased. All above results indicated that DWI and a variety of observation indicators from DWI were associated with cytotoxic edema and vascular edema of brain tissue, which was needed further study.

When there were no obvious clinical symptoms at early phase of acute cerebral ischemic disease, PWI showed abnormal changes. The changes of brain tissue after embolization were directly related to region cerebral perfusion pressure (rCPP) and local vascular circulation resistance (rCVR). The decreased rCBV and rCBF ratios on PWI were found in the acute infarction of cat brain by quantitative evaluation [22]. In the relatively low perfusion state, the compensatory dilatation of blood vessels made that rCBV was often increased higher than normal. At this stage, cerebral ischemia had not yet produced irreversible damage, ischemic neuronal cells had not yet died, and the application of NGF and other neuroprotective agents could effectively maintain their survival. In the case of severe hypoperfusion, the lesion continued to progress, cerebrovascular dilatation was decompensated, and cerebral vascular was collapsed, and then rCBV was decreased. When rCBV was decreased to 0, it indicated that neurons in the region had been completely dead, but also lost meaning of neuroprotective agents. Therefore, this study focused on observing and analyzing brain tissue region that were in relatively low perfusion in the ischemic region, and the region was “penumbra” on the image [23]. These regions were the key regions that NGF was applied to protect damaged neurons. These regions of NGF treatment group showed significant differences compared with the saline control group without NGF treatment.

PWI was used in the early diagnosis of ischemic cerebral infarction. 11 patients with T2WI-negative early cerebral infarction were received PWI scan, which found that 9 patients showed significant perfusion abnormalities, the affected side CBV was decreased, MTT was significantly increased, and the abnormal region was consistent to the abnormal region of CT and T2WI at final follow-up. PWI could reflect the minor hemodynamic changes in patients with ischemic cerebral infarction. It was usually sufficient to show insufficient perfusion before abnormalities found by routine MRI, thus contributed the qualitative diagnosis of early-stage cerebral infarction and the efficacy of early treatment.

## CONCLUSIONS

This study explored the use of MRI to evaluate the protective and repair effects of NGF on impaired neurons in acute cerebral infarction, and also demonstrated that DWI could reflect the role of neuroprotective agents and its mechanism. Although the observation indicators such as rCBV, MTT, rCVR, and rCPP were not deeply studied, in view of advantages that change of cerebral ischemia was found by DWI and PWI at the early stage, which had initially shown their great potential for neuroprotective drug role on cerebral ischemia and mechanism. These factors had some relevance with neurological protection mechanism of cerebral ischemia, and further studies were still needed.

## MATERIALS AND METHODS

### Experimental animals and grouping

27 healthy adult canines familiaris with weight 10-15 Kg and unlimited male and female were in this study, which were classified into 6 groups in accordance with the principle of completely random, including group A: NGF treatment for 6 hours (*n* = 5), group B: NGF treatment for 24 hours (*n* = 5), group C: NGF treatment for 7 days (*n* = 5), group D: NGF treatment for 3 months (*n* = 5), group E: normal saline control group (*n* = 5), group F: complete blank control group (*n* = 2). Each animal required surgery was selected right side cerebral hemisphere to establish middle cerebral artery occlusion (MCAO) infarct model. In the course of the experiment, if there were animal deaths because of animal deaths anesthesia accidents, individual differences in animals and other causes, the abnormal death of animal was no longer as the observation object and included in the statistics, and the other randomly selected animal was used for supplement.

### Establishment of animal model

The 3 ml medical grade injection silicone rubber (purchased from the Shanghai Rubber Products Institute), the substrate and the catalyst were fully mixed into glass tube with inner diameter of 1.1 mm, placed at 40°C for 2 hours, removed and cut into 8 mm [24]. The animal limbs were fixed, preserved skin was performed in the left side of hind leg, and fluid path was established and retained. 60 mg/Kg pentobarbital sodium was used for general anesthesia through intravenous fluid path. After the operation, the animal model of acute cerebral infarction was established, and the infarct time was calculated from injection of embolus as the starting point.

### Experimental treatment

After the animal model was successfully prepared, the first MRI scan was performed at 30 min after embolization. The site and direction of the administration were determined according to the lesion site of DWI image, the reference position and body position marker. Two trumpet retractor pull to both sides of soft tissue to expose skull, and a small hole was obtained by portable bone drill with a diameter of 2 mm drill installed. And 0.5 ml NGF (2000 BU, purchased from the Center for Geriatrics Prevention and Treatment of China Medical University) was injected by microinjector within 1 min for once. The saline control group was given only the same amount of saline, and the remaining procedure was the same as that of the treatment group.

### Neurologic impairment scores

The neurologic impairment scores were performed at 24 hours, 1 week and 3 months after operation according to method of Purdy PD [25]. The standard was shown in Supplementary Table 1.

### Morphological observation

According to the experimental requirements, animals of groups A-E were executed after MRI examination, the brain were taken as a specimen to observe the color and blood vessel of brain surface, brain edema and cross-sectional performance. 2 animals from groups A-D were taken for HE staining and light microscope, and 1 animal from group E was received pathology observation. 2 animals from groups A-D were taken for electron microscopy and synaptic counting, and 1 animal from group E was received ultrastructural observation. The vibration section was used for immunohistochemical staining specimens with 5-6 μm of slice thickness. And immunohistochemical SP technique was used to treat the section (primary antibody (Rabbit anti-human NGF antibody), secondary antibody (Biotin-labeled goat anti-rabbit antibody), triple antibody and DAB reagent were purchased from Beijing Zhongshan Biotechnology Co., Ltd.). The stained tissue slices were placed in clear water to terminate the color reaction. Alcohol was used for gradient dehydration, Hemo-De was used to be transparent, DPX was used to seal, and it was observed under light microscope (100 ×). The positive cells of the treatment and control groups were counted. Three identical ischemic brain tissue sections were taken from each canine, 10 field of view were randomly taken from cortex of each brain tissue section, and the arithmetic mean was calculated.

### MRI

MRI was performed by a Toshiba VISART/Hyper 1.5T magnetic resonance system (Toshiba medical system, Nasu, Japan), gradient field strength was 23mT / m, knee joint orthogonal (QD) was used to transmit and receiver coil, and image post-processing was performed on the SGI image workstation. . MRI examination was performed for NGF groups (group A-D) and the control groups (group E, F) at 0.5 hours, 3 hours, 6 hours, 12 hours, 24 hours, 7 days and 3 months after MCAO. The scanning sequence included spin echo T1 (SE-T1, T1WI), fast spin echo T2 (FSE-T2, T2WI), fluid attenuated inversion recovery (FLAIR), three-dimensional time-of-flight-magnetic resonance angiography (3DTOF-MRA), DWI, and PWI. The parameters of T1WI were TR/E 500/15, the average acquisition number of NAQ was 2.0, matrix was 256 × 320, filed of view (FOV) was16 cm × 16 cm, and layer thickness was 6 mm. The parameters of T2WI were TR/TE 4000/108, matrix was 256 × 400, and FOV was 16 cm × 16 cm. The parameters of FLAIR sequence were TR/TE 9000/120, TI300 ms, echo chain was 17, acquisition matrix was 192 × 256, and NAQ was 2.0. DWI used a single excitation SE-EPI sequence, respectively, in the pe, ro, ss three directions to exert a diffusion-sensitive gradient, obtaining three groups of diffusion weight images, the direction b = 1000 sec/mm^2^. The TR was 6000 ms, the TE was 130 ms, layer thickness was 6mm, acquisition matrix was128 × 128, FOV was 25 cm × 32 cm, and reconstruction matrix was 256 × 256. PWI used two stimulated FE-EPI sequences, TR was 2000 ms, TE was 40 ms, reversal angle was 90 degrees, and the setting of layers were same as DWI, T2WI and FLAIR.

### Image postprocessing of DWI

DWI images could be processed by workstation SGI O2, images post processing software to obtain the ADC map. The ADC signal strength was the ADC value in diffusion direction, and could be calculated by follow formula: ADC = -ln (S1/S0)/(b0-b1); ADCR = ADC value of ischemic region / ADC value of contralateral region; HLV% = (area of abnormal range / hemispherical area of abnormal area) x 100%. Where ADC was the apparent diffusion coefficient, b was the diffusion sensitivity coefficient, S was the signal intensity under a certain diffusion coefficient, where S0 and S1 were the signal intensities of pixel for DWI image taking b0 =0 and b1=1000, respectively. ADCR was the ADC ratio. HLV is hemispheric abnormal volume ratio.

### Image postprocessing of PWI

After the PWI scan, the perfusion curve of the ROI was obtained from dynamic analysis by workstation SGI O2, images post processing software. The region relative cerebral blood volume (rCBV) values and mean transit time (MTT) were calculated from the PWI perfusion curve, MTT could be calculated by follow formula: rCBV=(t∫R*∫dt), where t represented the time (seconds); ΔR*=1/ΔT2*=−ln(Sn/S1)/TE, where Sn was signal strength of the lesion in the moment of "n", TE was the echo time. Through the above formula, under the curve area could be calculated. The region cerebral blood flow velocity (rCBF) was (ΔR * dt), and MTT was rCBV/rCBF.

### Statistical analysis

SPSS 16.0 software was used for statistical analyses. Differences between two groups were tested by t-test and Mann Whitney test, and one-way ANOVA was used for comparison among different groups. *P*<0.05 was consider to be statistically significant.

## SUPPLEMENTARY MATERIALS FIGURES AND TABLES



## Abbreviations

(NGF): nerve growth factor
(MCAO): middle cerebral artery occlusion
(MRI): magnetic resonance imaging
(DWI): diffusion-weighted imaging
(PWI): perfusion-weighted imaging
(SE-T1, T1WI): spin echo T1
(FSE-T2, T2WI): fast spin echo T2
(FLAIR): fluid attenuated inversion recovery
(3DTOF-MRA): three-dimensional time-of-flight-magnetic resonance angiography
(rCBV): region relative cerebral blood volume
(MTT): mean transit time
(rCBF): The region cerebral blood flow velocity
(rCPP): region cerebral perfusion pressure
(rCVR): region local vascular circulation resistance
