# Supplementary material for: Therapeutic effect of nerve growth factor on canine cerebral infarction evaluated by MRI
Source: Oncotarget. 2017 Dec 16;9(3):3741–51. doi: 10.18632/oncotarget.23345 (PMC5790496; doi:10.18632/oncotarget.23345)
Supplement: Supplementary file 1 [file oncotarget-09-3741-s001.pdf]

Therapeutic effect of nerve growth factor on canine cerebral infarction evaluated by MRI

SUPPLEMENTARY MATERIALS

Supplementary Table 1: Neurological deficit score assessment table of canine stroke model

Performance	Scores
Behavior	
No obvious defects and normal neurological function	1
Tilted to one side, and not rely on external forces to restore their own balance	2
Only rely on external forces to stand	3
Only hemiplegia and awake, but still cannot stand with external force	4
Coma or death, cannot be measured	4
**Consciousness**	
Sober and normal response	1
Awake, reduced or diminished consciousness	2
Awake, severely impaired consciousness	3
Coma or death	4
**Head rotation**	
No visible activity on the head	0
The head is mandatory or tends to lean toward the infarct side	1
Cannot lift the head, coma or death	1
**Circle**	
No obvious circular motion	0
The route is a circle	1
Cannot walk or die	1
**Hemianopia**	
Not show positive test results	0
When walking hit the ipsilateral wall	1
Repeated front tests showed significant asymmetric results	1
Cannot be tested for diminished consciousness or death	1
**Total scores**	
Minimal (completely normal and undamaged animals)	2
Maximum (coma or death)	11

Supplementary Table 2: General observation of different group at different ischemic time

Groups	Ischemic time	Brain surface color	Brain surface blood vessels	Midline displacement situation	Cortex and medulla interface
B	24 hours	Light red	Thicken, increased	Relatively obvious	Lack of clear
E	24 hours	Pale	Thicken, increased	Very obvious	Blurry
D	3 months	Normal	Normal	None	Relatively clear
E	3 months	Normal	Normal	None	Relatively blurry

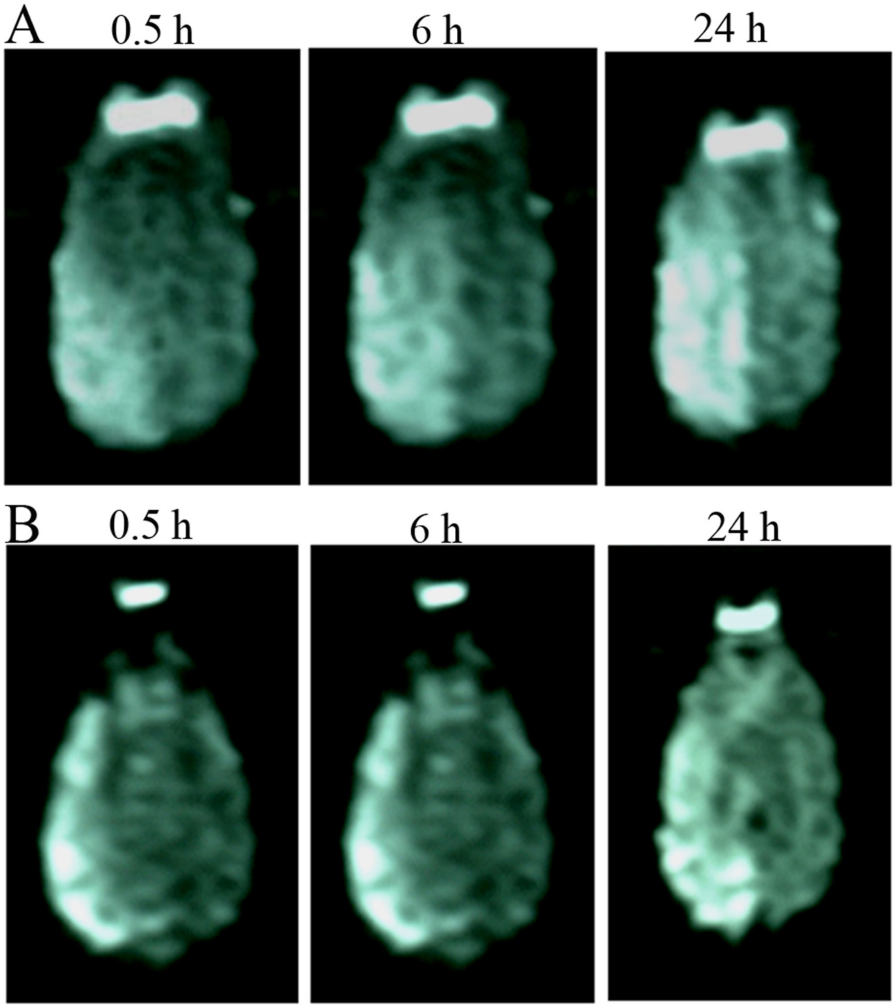

Supplementary Figure 1:The DWI images of canines brain after stroke. (**A**) After stroke, the signal intensity of lesion at 0.5 h, 6 h and 24 h in those without treating canines from group E (normal saline control); (**B**) After stroke, the signal intensity of lesion at 0.5 h, 6 h and 24 h in those treated canines from group D (NGF treatment for 3 months).

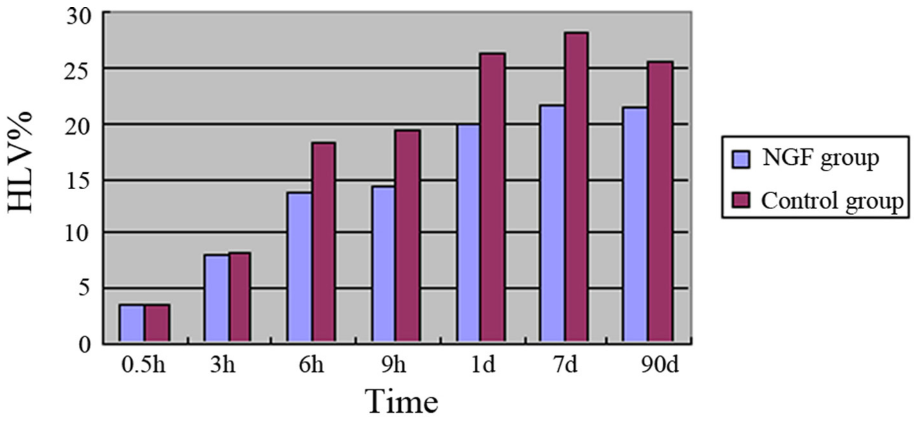

Supplementary Figure 2: HLV% of lesion between group D (NGF treatment for 3 months) and group E (normal saline control) at different time.

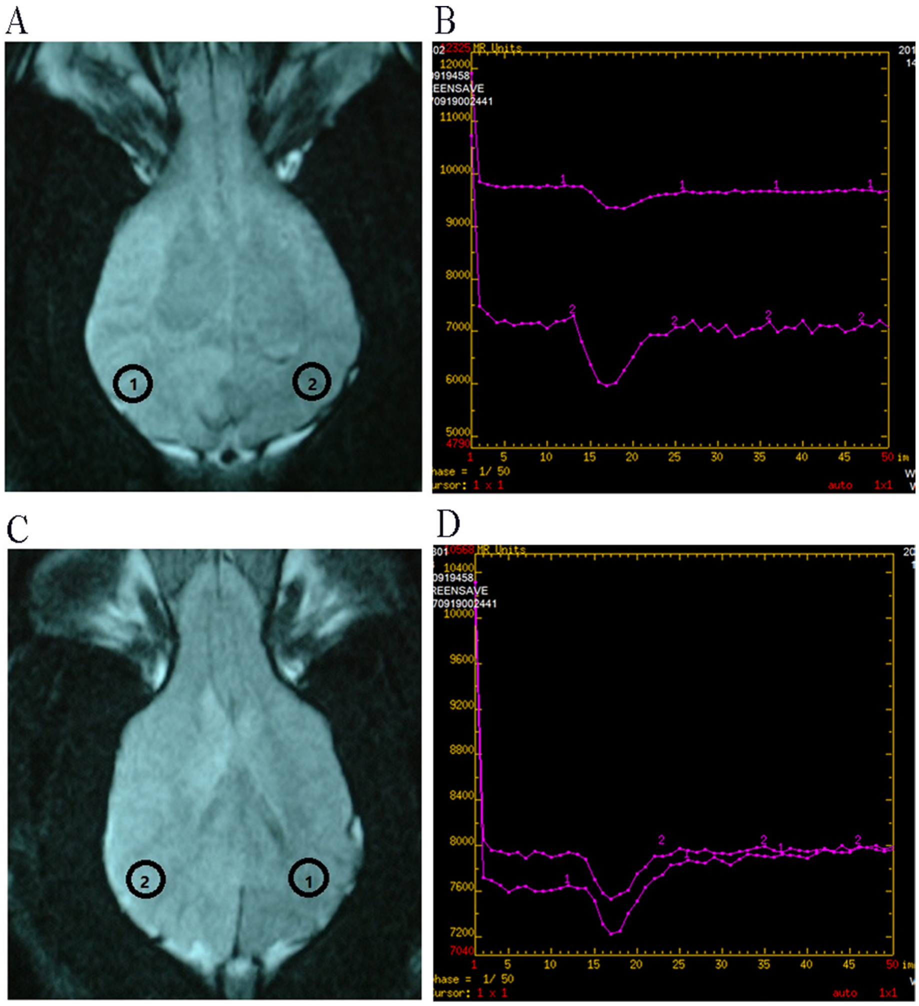

Supplementary Figure 3: Perfusion curve of infarct focus group E (normal saline control) and group B (NGF treatment for 24 hours). (**A**) Settings of interest area in group E; (**B**) The perfusion curve of group E, line 1 was the perfusion curve of the infarct focus, line 2 was the perfusion curve of the contralateral normal tissue; (**C**) Settings of interest area in group B; (**D**) The perfusion curve of group B, line 1 was the perfusion curve of the contralateral normal tissue, line 2 was the perfusion curve of the infarct focus.

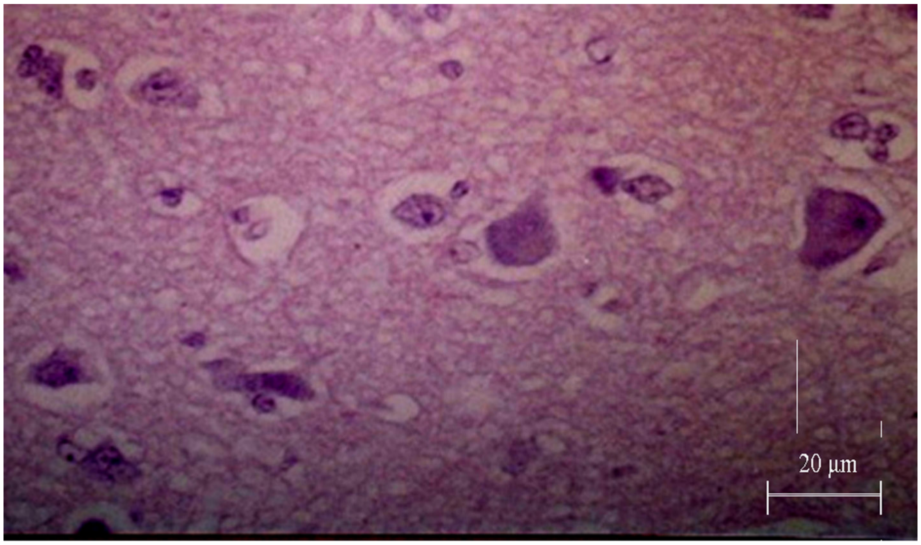

Supplementary Figure 4: Pathology changing of infarction tissue from group A (NGF treatment for 6 hours).

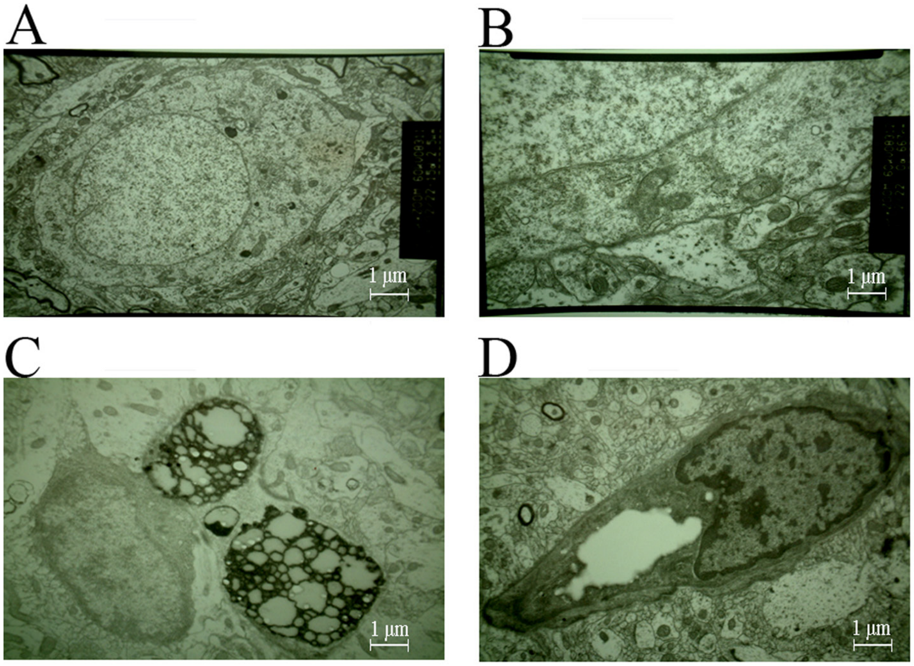

Supplementary Figure 5: (**A**) The normal ultrastructure neuron of canines' brain (×4000); (**B**) The normal ultrastructure neuron of canines' brain (×20000); (**C**) Many lipofascin particles could been seen in cytoplasm or in dendrites (×15000); (**D**) The cytoplasm or in dendrites (×15000).
